# Synergistic effect and mechanism of meropenem with ciprofloxacin against carbapenem-resistant *Acinetobacter baumannii*

**DOI:** 10.3389/fphar.2025.1534155

**Published:** 2025-05-15

**Authors:** Ying Feng, Xu Chen, Yu Sun, Tingting Guo, Feng Wu, Feng Jin, Jun Zhou

**Affiliations:** ^1^ Department of Respiratory and Critical Care Medicine, Affiliated Hospital of Yangzhou University, Yangzhou University, Yangzhou, China; ^2^ Department of Microbiology, Medical College, Yangzhou University, Yangzhou, China

**Keywords:** carbapenem-resistant *Acinetobacter baumannii* (CRAB), meropenem, ciprofloxacin, biofilm formation, transcriptomics

## Abstract

**Introduction:**

Acinetobacter baumannii has been recognized as a major public health concern, and carbapenems have been considered the first-line treatment for Acinetobacter baumannii infections. However, their widespread and prolonged use has led to the emergence of carbapenem-resistant Acinetobacter baumannii (CRAB), which has become a significant nosocomial pathogen. The control and treatment of CRAB infections have become a global challenge.

**Methods:**

Logistic regression was applied to investigate the clinical characteristics and prognostic risk factors of CRAB infections. Multilocus sequence typing (MLST) of three clinical CRAB isolates was carried out to determine their genotype. The antibacterial efficacy of meropenem combined with ciprofloxacin was evaluated using checkerboard and growth curve assays. Transcriptomics analysis was futher used to analysis the molecular mechanism related to the combination treatment.

**Results:**

Logistic regression analysis identified the duration of antibiotic use, glucocorticoid application, C-reactive protein levels, and creatine kinase-MB levels as independent risk factors for poor prognosis in CRAB patients. Multilocus sequence typing of three CRAB isolates revealed that all isolates were ST208 genotype. Checkerboard and Growth Curve assays demonstrated that the combination of ciprofloxacin and meropenem had significant bactericidal effects on CRAB, outer membrane permeability, membrane potential, and reactive oxygen species generation. Transcriptomic analysis revealed that the combination inhibited efflux pump function, reduced iron uptake, and impacted metabolic pathways, membrane protein synthesis, and stress responses, thereby enhancing bacterial killing.

**Discussion:**

The findings from this study underscore the synergistic effect of ciprofloxacin and meropenem not only offers a potential alternative treatment strategy but also highlights the importance of combination therapy in overcoming antibiotic resistance, which pose a significant threat to public health.

## Introduction

*Acinetobacter baumannii* (AB) is a Gram-negative, aerobic, non-fermentative bacterium primarily associated with hospital-related infections (Maragakis and Perl, 2008). It can cause respiratory tract infections, bloodstream infections, and more (Sievert et al., 2013). When immune function is compromised or invasive procedures such as intubation, catheter insertion, or surgery are performed, AB can breach mucosal barriers, enter the bloodstream, or spread to other tissues, leading to infection (Iovleva et al., 2022). AB has strong antibiotic resistance and clonal spread capabilities (Garnacho-Montero and Amaya-Villar, 2010). In fact, multidrug-resistant (MDR), extensively drug-resistant (XDR), and pan-drug-resistant (PDR) AB strains have been reported globally (Weinberg et al., 2020). In China, AB has become one of the most important pathogens in nosocomial infections (Dijkshoorn et al., 2007). Multidrug-resistant *Acinetobacter baumannii* (MDRAB) refers to AB strains resistant to multiple classes of antibiotics, which are more likely to infect immunocompromised or underlying disease patients (Paterson and Doi, 2007).

In the past, infections caused by MDRAB have significantly increased (Munoz-Price et al., 2013). Although carbapenems were once considered the first choice for treating MDRAB infections, the rapid rise of carbapenem resistance globally in recent years has raised widespread concern (Dinc et al., 2013). In Asia, Eastern Europe, and Latin America, carbapenem resistance rates have exceeded 30%–90% (O’Donnell et al., 2021; Shields et al., 2023). Additionally, carbapenems are considered one of the main risk factors for the emergence and spread of MDRAB (Garnacho-Montero et al., 2015). The natural and acquired resistance of AB limits the treatment options for CRAB infections (Shields et al., 2023). CRAB strains often exhibit extensive drug resistance (XDR), meaning resistance to all antibiotics except polymyxins and tigecycline. However, CRAB strains resistant to polymyxins and/or tigecycline have been increasingly reported, significantly increasing the difficulty of clinical treatment (Bell and Park, 2023; Zhao et al., 2023). The high incidence of pan-drug resistance in many clinical isolates is attributed to the complex resistance mechanisms of this pathogen, including biofilm formation, active efflux pumps, and more (Antimicrobial Resistance et al., 2022). As a result, CRAB has become one of the most challenging nosocomial pathogens (Tiku et al., 2021). The main mechanism of carbapenem resistance in AB is the production of carbapenemases (classes D, A, and B) (Bartal et al., 2022). In CRAB, the most common are OXA-type β-lactamases, which are divided into multiple subgroups, with over 400 OXA-type enzymes identified (Evans et al., 2013). Previous studies have shown that CRAB can form biofilms on various material surfaces, including medical devices, rubber, and endotracheal tubes (Greene et al., 2016). Biofilm formation increases the risk of device-associated infections (Pour et al., 2011). Outer membrane proteins (OMPs) are a class of integral membrane proteins embedded in the outer membrane, with OmpA being one of the most abundant porins in the CRAB outer membrane (Uppalapati et al., 2020). Studies have shown that OmpA not only participates in the process of expelling antibiotics from the periplasmic space through the outer membrane but also works in conjunction with the inner membrane efflux system (Confer and Ayalew, 2013). Additionally, OmpA promotes biofilm formation, further enhancing the survival and persistence of AB, and its overexpression is closely associated with a significant increase in pneumonia, bacteremia, and mortality in patients (Sanchez-Encinales et al., 2017). Similarly, the AdeABC efflux pump is highly expressed in CRAB, which is closely related to its resistance to carbapenem antibiotics. Studies have shown that the AdeABC effluxau pump significantly reduces the effective concentration of antibiotics within the cell by expelling them from the cell, thereby enhancing CRAB resistance (Paul et al., 2022; Tamma et al., 2022). In response to these phenomena, researchers have focused on developing various combination therapy regimens, including minocycline/tigecycline and colistin/tigecycline (Castanheira et al., 2014; Menegucci et al., 2019). Although these alternative regimens have shown some efficacy in treating nosocomial infections, their effectiveness remains limited.

In this study, data were collected from 137 patients with carbapenem-resistant *A. baumannii* (CRAB) infections and 131 patients with non-carbapenem-resistant *A. baumannii* infections admitted to Yangzhou University Affiliated Hospital from July 2021 to September 2022. A series of analyses were conducted on clinical resistance patterns, biochemical characteristics, and prognostic risk factors. The aim is to provide theoretical insights for clinical antimicrobial strategies and improve the poor prognosis of CRAB patients. Due to the different resistance mechanisms of AB to carbapenems and fluoroquinolones, it is unclear whether the combined use of these two drugs can effectively kill CRAB and its specific mechanisms. Thus, three CRAB strains with strong biofilm formation were selected, and the *in vitro* antibacterial activity of meropenem combined with ciprofloxacin against CRAB was studied.

## Materials and methods

### Selection of study subjects

This study selected clinical data from 137 CRAB patients and 131 AB patients hospitalized at the Affiliated Hospital of Yangzhou University from July 2021 to September 2022. The age range of all patients was 16–94 years, with an average age of 69 years; 174 were male (64.9%) and 94 were female (35.1%); 196 patients (73.1%) were over 60 years old. All strains were isolated from sputum specimens of hospitalized patients.

### Inclusion criteria

New or progressive radiographic pulmonary infiltrates appearing 48 h after admission, accompanied by at least two clinical infection symptoms (temperature >38°C or <35.5°C, leukocytosis or leukopenia, purulent secretions). For elderly patients, clinical manifestations may be atypical, such as altered mental status, anorexia, fatigue, or lack of fever. Inclusion criteria also included bacteriological test results.

### Exclusion criteria

Patients were excluded if they met any of the following conditions: (i) AB detected within 48 h of admission; (ii) age under 16 years; (iii) incomplete clinical data; (iv) duplicate specimens from the same or different sites of the same patient; (v) bacterial colonization without clinical symptoms, radiographic, or laboratory abnormalities.

### Detection of β-lactamase genes in *A baumannii* isolates

We screened for common β-lactamase genes (blaPER, blaSHV, blaIMP, blaVIM, blaSIM, blaNDM-1, blaAmpC, blaOXA-23, blaOXA-24, blaOXA-51, blaOXA-143, blaOXA-58), and sent the primers to Nanjing GenScript Biotechnology Co., Ltd. for synthesis. PCR detection was performed on CRAB 27 and CRAB 49. The final reaction volume for PCR amplification was 20 μL, containing 1 μL of template, 10 μL of Mix, 1 μL each of forward and reverse primers, and 7 μL of ddH_2_O. The amplification conditions were as follows: pre-denaturation at 95°C for 5 min, denaturation at 95°C for 30 s, annealing for 30 s, extension at 72°C for 1 min, and 35 cycles. The PCR products were subjected to agarose gel electrophoresis and photographed for documentation. The primer sequences are shown in Table 1.

**TABLE 1:** Primers used in this study.

Primers	Sequence (5′ to 3′)	Size (bp)	Annealing temperature
blaOXA-23 blaOXA-24 blaOXA-51 blaOXA-58	F: GATGTGTCATAGTATTCGTCG R: TCACAACAACTAAAAGCACTG F: ATGAAAAAATTTATACTTCCTATATTCAGC R: TTAAATGATTCCAAGATTTTCTAGC F: TAATGCTTTGATCGGCCTTG R: TGGATTCGACTTCATCTTGG F: AAGTATTGGGGCTTGTGCTG R: CCCCTCTGCGCTCTACATAC	1,067 828 353 599	53°C 54°C 54°C 57°C
blaOXA-143	F: AACCTGACACGAGCACATAC R: CCAGGCATTCCTTGCTTCAT	569	56°C
BlaVIM	F: GATGTGTTTGGTCGCATA R: CGAATGCGCAGCACCAG	390	54°C
BlaSHV BlaSIM blaNDM-1 blaPER blaAmpC blaIMP	R: ATTTGTCGCTTCTTTACTCGC F: TTTATGGCGTTACCTTTGACC R: TACAAGGGATTCGGCATCG F: TAATGGCCTGTTCCCATGTG R: GAATGTCTGGCAGCACACTT F: TTGGCCTTGCTGTCCTTGAT R; ATGAATGTCATTATAAAAGC F: AATTTGGGCTTAGGGCAGAA R: CGGGCAATACACCAAAAGAC F: CCTTAATGCGCTCTTCATTTGG R: GGAATACAGTGGCTTAACTCTC F: CCAAACCACTAGGTTATCT	1,051 570 1067 925 1,049 188	54°C 55°C 57°C 50°C 56°C 53°C

### Bacteria culture and biofilm screening

Bacteria culture and isolation were performed according to the standards of the “National Clinical Laboratory Procedures” (fourth edition). For the same site of the same patient, only the first isolated strain was analyzed. Strain identification was conducted using the Vitek 2 Compact automated microbiology analyzer and Vitek MS automated mass spectrometer from bioMérieux, France. Five strains isolated from clinical samples were inoculated onto LB agar plates and isolated using the four-quadrant streaking method. The inoculated plates were incubated overnight at 37°C, and uniform single colonies were selected using an inoculating loop. The selected single colonies were inoculated into 2 mL of LB medium and cultured overnight at 37°C and 200 rpm in a shaker. The cultured bacterial solution was mixed with 40% glycerol at a 1:1 ratio and stored at −80°C for subsequent experiments. Next, the stored bacterial solution was inoculated into 1 mL of MH broth at a 1:100 ratio and cultured until the OD600 nm value reached 0.5. Then, 10 µL of the bacterial solution with an OD600 nm of 0.5 was added to 990 µL of MH broth for a 1:100 dilution. The diluted bacterial solution (200 µL) was added to a 96-well microplate, with three replicates for each strain and 200 µL of MH medium as a blank control. The microplate was incubated statically at 37°C for 18 h. After incubation, the supernatant was discarded, and the wells were washed twice with PBS, stained with 0.4% crystal violet for 30 min, and washed twice again with PBS. Finally, the dye was eluted with 95% ethanol, and the absorbance was measured at OD590 nm using a microplate reader.

### Antibiotic susceptibility testing

Single colonies were inoculated into 10 mL of MH broth and cultured at 37°C and 200 rpm until the OD600 nm reached 0.5. The bacterial solution was then diluted 100-fold to a final concentration of 1 × 10^6^ CFU/mL. Different concentrations of antibiotic solutions (0.5, 1, 2, 4, 8, 16, 32, 64 μg/mL) were prepared, and 100 µL of the corresponding concentration of antibiotic was added to each test well. Another 100 µL of the diluted bacterial solution was added to each well, with a negative control well containing 200 µL of MH medium. After thorough mixing, the plates were incubated at 37°C for 18 h. After incubation, the OD600 nm value was measured using a microplate reader. The minimum inhibitory concentration (MIC) of the antibiotic was defined as the lowest concentration that inhibited bacterial growth. Antibiotic susceptibility results were determined according to the 2021 Clinical and Laboratory Standards Institute (CLSI) guidelines and classified as resistant, intermediate, or sensitive. Quality control strains included *Escherichia coli* ATCC 25922 and *Pseudomonas aeruginosa* ATCC 27853. All experimental operations strictly followed standard operating procedures. All experiments were repeated three times to ensure reproducibility and reliability of the results.

### Data collection

Complete medical records of CRAB patients meeting the inclusion criteria were collected, including the following: (i) Basic patient information: gender, age, history of diabetes, history of invasive procedures, glucocorticoid use, length of hospital stay, length of ICU stay, duration of mechanical ventilation, antibiotic use; (ii) Clinical indicators and antibiotic susceptibility results; (iii) Hospital outcomes: outcomes were classified as improvement (including cure and clinical and/or laboratory and radiographic improvement) and poor prognosis (including death and non-recovery).

### Multilocus sequence typing (MLST)

PCR amplification of seven housekeeping genes (gltA, gyrB, gdhB, recA, cpn60, gpi, rpoD) was performed using internationally recognized primers. The amplification products were sequenced by Nanjing GenScript Biotechnology Co., Ltd. According to the Oxford MLST scheme, MLST typing analysis was performed on strains AB1, AB2, and AB3. The obtained gene sequences were uploaded to the *Acinetobacter baumannii* PubMLST database (http://pubmlst.org/abaumannii/) to determine their sequence type (ST) by comparison with existing sequences in the database. The total volume of the PCR reaction system was 25 μL, including 1 µL of primer, 1 µL of template, 12.5 µL of PCR Mix, and 9.5 µL of ddH2O. The PCR amplification conditions were set as follows: pre-denaturation at 94°C for 5 min; denaturation at 94°C for 30 s, annealing for 30 s, extension at 72°C for 1 min, for a total of 30 cycles; final extension at 72°C for 10 min. Primer sequences and annealing temperatures are detailed in Supplementary Table S1. All experiments were performed with three technical replicates to ensure the reproducibility and reliability of the results.

### Checkerboard broth microdilution and the growth curve assays

To evaluate the *in vitro* antibacterial activity of antibiotic combinations against CRAB, a checkerboard broth microdilution assay was first performed. Based on the results of the previous antibiotic susceptibility tests, the MICs of the two antibiotics were determined, and their concentrations were further diluted to one to two times the MIC. According to the two-fold dilution method, the two antibiotics were diluted along the vertical and horizontal coordinates of the 96-well plate, with 50 µL of the corresponding concentration of antibiotic MH broth added to each well. After overnight culture at 37°C, single colonies were transferred to 5 mL of fresh MH broth and cultured until the OD590 nm reached 0.5 (1 × 10^8^ CFU/mL), then diluted 100-fold to a final concentration of 1 × 10^6^ CFU/mL. Each well was inoculated with 100 µL of the diluted bacterial solution and mixed with 100 µL of MH broth containing antibiotics, with negative and blank controls set. After mixing, the plates were incubated at 37°C for 18 h, and the optical density (OD) at 600 nm was measured using a microplate reader. The bacterial synergistic inhibition rate was calculated using the following formula: Bacterial synergistic inhibition rate (%) = [(OD positive control group − OD negative control group) − (OD sample - OD negative control group)]/(OD positive control group − OD negative control group) × 100%. The fractional inhibitory concentration index (FICI) of the combined drugs was calculated using the following formula: FICI = MIC meropenem (combination)/MIC meropenem (single) + MIC ciprofloxacin (combination)/MIC ciprofloxacin (single). FICI ≤0.5, synergy; 0.5≤ FICI ≤4, no interaction; FICI >4, antagonism (Vidaillac et al., 2012). Additionally, the growth curve assay was performed on the CRAB2 strain. After streaking single colonies using the quadrant method, they were cultured overnight at 37°C and transferred to 5 mL of fresh MH broth at a 1:100 ratio, cultured until the OD590 nm was 0.5 (1 × 10^8^ CFU/mL), and then diluted to 1 × 10^6^ CFU/mL. Subsequently, the two antibiotics were added to the bacterial solution alone or in combination, with no antibiotics added as the control group. The cultures were incubated at 37°C and 200 rpm, and 200 µL samples were taken every 2 h, and the OD600 nm was measured using a microplate reader to monitor bacterial growth (YOURASSOWSKY et al., 1985).

### Data analysis

Analysis of CRAB resistance, intermediate resistance, and sensitivity was performed using SPSS 26.0 software for statistical analysis. For quantitative data that follow a normal distribution or an approximate normal distribution, they are presented as mean ± standard deviation. Independent-samples t-test or one-way ANOVA (depending on the number of groups) is used for analysis. If the data do not meet the requirements of normal distribution or homogeneity of variance, they are described by median/interquartile range. The Mann-Whitney U test for two independent samples or the Kruskal–Wallis test for multiple independent samples is used for result analysis. Categorical variables or ranked data are described using percentages. The chi-square test or Fisher’s exact probability method is used for between-group difference analysis.

### Transcriptomic analysis

The AB-27 strain of CRAB was selected for transcriptomic analysis. After culturing AB-27 in Mueller-Hinton broth (MHB) to the early logarithmic growth phase, the strain was treated with a combination of ciprofloxacin and meropenem. Following RNA quality assessment, cDNA synthesis, fragmentation, and sequencing were performed. The cDNA library was then amplified and sequenced using the Illumina HiSeq™ platform. Technical services and subsequent bioinformatics analysis were provided by Guangzhou Gene *Denovo* Biotechnology Co., Ltd. Differentially expressed genes (DEGs) were identified through comparative analysis of RNA-Seq data. Differential expression analysis was conducted using the DESeq2 package, and genes with significant differences were selected based on the predefined thresholds (P < 0.05, |log_2_FoldChange| > 1). For the selected DEGs, Gene Ontology (GO) enrichment analysis was performed using the R package clusterProfiler. The GO annotations were categorized into three main terms: Biological Process (BP), Molecular Function (MF), and Cellular Component (CC). The significance of each GO term was calculated using Fisher’s exact test, followed by multiple testing correction using the Benjamini–Hochberg method. GO terms with a P-value <0.05 were considered significantly enriched. KEGG pathway enrichment analysis was performed on the DEGs using the KEGG database. KEGG enrichment analysis was conducted using the R package clusterProfiler, which calculates the enrichment of DEGs across various KEGG pathways using Fisher’s exact test, followed by False Discovery Rate (FDR) correction for P-values. Pathways with a P-value <0.05 were considered significantly enriched.

## Results

### Distribution of CRAB strains

In this study, 137 carbapenem-resistant *A. baumannii* (CRAB) strains were isolated from sputum samples. These strains were primarily obtained from the Intensive Care Unit (ICU) (103 cases), the Department of Respiratory Medicine (12 cases), and the Department of Neurosurgery (11 cases), accounting for 75.2%, 8.8%, and 8.0% of the total, respectively (Supplementary Table S2).

### Antibiotic resistance of CRAB strains

In this study, carbapenem resistant *Acinetobacter baumannii* strains were selected for research. Clinical antimicrobial susceptibility tests on 137 CRAB strains against 24 antimicrobial agents were conducted. Results showed that among β-lactam antibiotics, resistance rate to carbapenem antibiotics was the highest, reaching 99.27%. This was followed by third-generation cephalosporins, with a resistance rate exceeding 96%. The resistance rates to broad-spectrum penicillins (such as piperacillin and ticarcillin) were also high, exceeding 95%.CRAB exhibited moderate resistance to tigecycline, with a resistance rate of 42.33%, while minocycline still showed good antibacterial activity against CRAB, with a resistance rate of 25.54% (Supplementary Table S3).

### Comparison of clinical characteristics between CRAB and AB

To compare the clinical characteristics between the CRAB group and the AB group, we conducted a comparative analysis of multiple parameters in both groups of patients. These parameters included the number of invasive procedures, length of hospital stay, length of ICU stay, duration of mechanical ventilation, types and duration of antibiotic use, conjugated bilirubin levels, alanine aminotransferase (ALT) levels, aspartate aminotransferase (AST) levels, urea levels, and the number of unresolved cases. We found that the CRAB group had higher values in all these indicators compared to the AB group, with statistically significant differences between the two groups (*P* < 0.05). The number of patients undergoing invasive procedures was 129 in the CRAB group and 111 in the AB group, accounting for 94.16% and 84.73%, respectively. The length of hospital stay was 21 ± 11 days for the CRAB group and 17 ± 9 days for the AB group. The length of ICU stay was 13 ± 9 days for the CRAB group and 5 ± 7 days for the AB group. The duration of mechanical ventilation was 9 ± 8 days for the CRAB group and 3 ± 6 days for the AB group. The number of types of antibiotics used was 4 ± 7 in the CRAB group and 2 ± 1 in the AB group. The duration of antibiotic use was 18 ± 9 days in the CRAB group and 13 ± 7 days in the AB group. The conjugated bilirubin level was 6.32 ± 22.40 μmol/L in the CRAB group and 3.69 ± 14.68 μmol/L in the AB group. The average alanine aminotransferase (ALT) level was 36.00 U/L in the CRAB group and 23.00 U/L in the AB group. The average aspartate aminotransferase (AST) level was 28.00 U/L in the CRAB group and 41.00 U/L in the AB group. The urea level was 11.19 ± 7.00 mmol/L in the CRAB group and 8.41 ± 5.67 mmol/L in the AB group. The number of unresolved cases was 53 in the CRAB group and 25 in the AB group, accounting for 38.69% and 19.08%, respectively (Supplementary Table S4).

### Clinical characteristics of poor prognosis in CRAB patients

To elucidate the prognosis of patients with CRAB infection, we compared multiple parameters between the non-improvement group and the improvement group. The results showed that the non-improvement group had higher values in ICU stay days, mechanical ventilation days, white blood cell count, neutrophil count, C-reactive protein level, procalcitonin level, conjugated bilirubin, unconjugated bilirubin, aspartate aminotransferase, urea, creatinine, and creatine kinase-MB compared to the improvement group, with statistically significant differences between the two groups (*P* < 0.05). Specifically, the ICU stay days were 15 ± 11 days in the non-improvement group and 11 ± 8 days in the improvement group; mechanical ventilation days were 11 ± 9 days in the non-improvement group and 8 ± 8 days in the improvement group; white blood cell count was 12.17 ± 5.19 × 10^9^/L in the non-improvement group and 9.82 ± 3.53 × 10^9^/L in the improvement group; neutrophil count was 11.37 ± 7.00 × 10^9^/L in the non-improvement group and 8.05 ± 3.20 × 10^9^/L in the improvement group; C-reactive protein level was 102.19 ± 77.72 mg/L in the non-improvement group and 50.09 ± 39.31 mg/L in the improvement group; procalcitonin level was 4.49 ± 8.57 ng/L in the non-improvement group and 0.68 ± 1.64 ng/L in the improvement group; conjugated bilirubin was 14.60 ± 34.45 μmol/L in the non-improvement group and 1.10 ± 2.67 μmol/L in the improvement group; unconjugated bilirubin was 16.06 ± 12.83 μmol/L in the non-improvement group and 10.24 ± 6.61 μmol/L in the improvement group; aspartate aminotransferase average level was 53.00 U/L in the non-improvement group and 36.00 U/L in the improvement group; urea was 13.84 ± 8.40 mmol/L in the non-improvement group and 9.52 ± 5.36 mmol/L in the improvement group; creatinine was 126.59 ± 94.72 μmol/L in the non-improvement group and 83.21 ± 75.63 μmol/L in the improvement group; creatine kinase-MB was 19.45 ± 26.03 U/L in the non-improvement group and 7.64 ± 6.40 U/L in the improvement group. These data indicate that the clinical manifestations and laboratory indicators of patients in the non-improvement group were significantly worse than those in the improvement group (Supplementary Table S5).

### Risk factors for prognosis of CRAB infection

**FIGURE 1:**
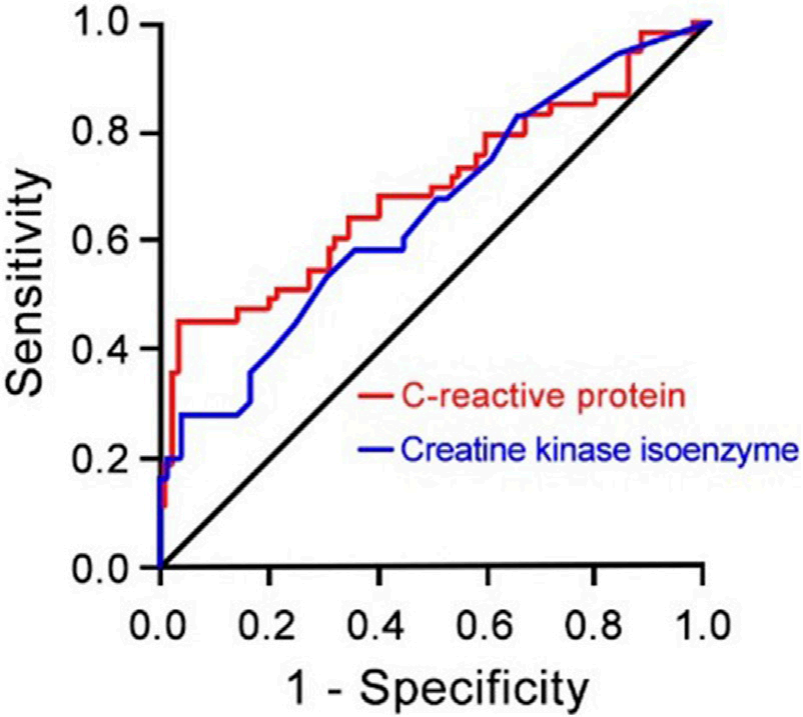
Receiver Operating Characteristic (ROC) Curve for C-reactive Protein (red lines) and Creatine Kinase Isoenzyme (blue lines) as Risk Factors for Prognosis of CRAB Infection (N = 137) compared to AB patients (N = 131). CRAB, carbapenem-resistant *Acinetobacter baumannii*; AB, *Acinetobacter baumannii*.

The results of the logistic multivariate regression analysis showed that the duration of antibiotic use, application of glucocorticoids, C-reactive protein levels, and creatine kinase isoenzyme levels were independent risk factors for poor prognosis in CRAB patients (*P* < 0.05) (Table 2). Additionally, C-reactive protein and creatine kinase isoenzyme levels have certain predictive value in forecasting poor prognosis in CRAB patients, with an AUC of 0.69 for C-reactive protein and an AUC of 0.64 for creatine kinase isoenzyme (Figure 1).

**TABLE 2:** Risk factors for prognosis of CRAB infection.

Variable	B value	Standard error	Wald value	P value	OR	Confidence interval
Duration of antibiotic use	−0.11	0.054	4.09	0.043	0.896	0.806–0.997
Application of glucocorticoids	1.554	0.664	5.48	0.019	4.73	1.288–17.372
C-reactive protein	0.012	0.005	5.268	0.022	1.012	1.002–1.023
Creatine kinase isoenzyme	0.063	0.031	4.293	0.038	1.065	1.003–1.131

### Biofilm strength and antibiotic susceptibility testing

**FIGURE 2:**
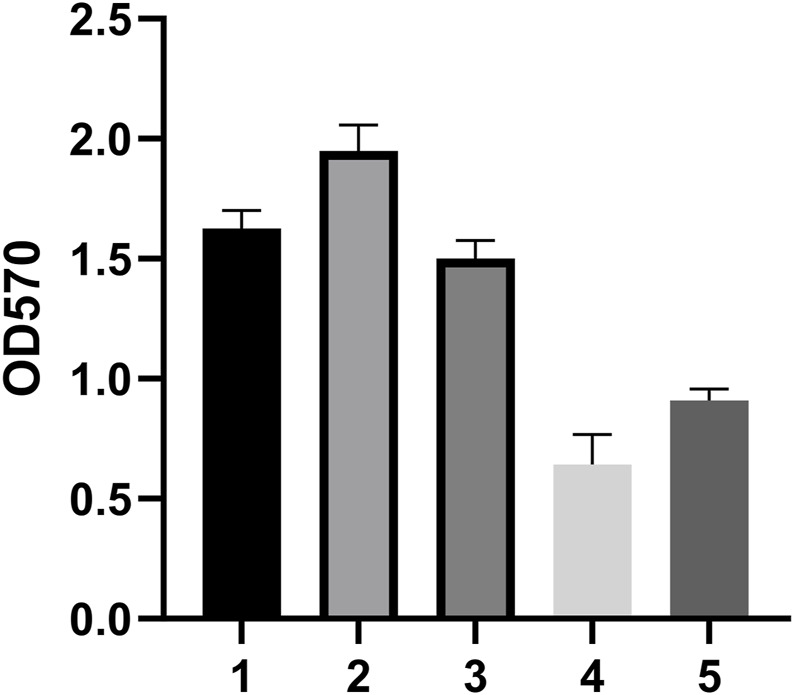
Biofilm Formation Ability of 5 Clinically Isolated CRAB Strains. The data are expressed as mean ± SD derived from three biological replicates. The Kruskal–Wallis test was utilized to assess differences in fluorescence alterations among the four groups, followed by the Mann-Whitney U test for pairwise comparisons between groups. **(A–D)** Bars sharing the same annotation indicate no statistically significant differences between groups, while bars with different annotations denote statistically significant differences between groups.

We tested the biofilm formation ability of five CRAB strains, with results reflected by OD590 nm values. The results indicated that strain 2 had the strongest biofilm formation ability, followed by strains 1 and 3 (Figure 2). Therefore, we selected these three strong biofilm-forming CRAB strains for further antibiotic susceptibility testing. We found that all strains exhibited high levels of resistance (MIC ≥64 μg/mL) to meropenem (MEM), imipenem (IPM), gentamicin (GEN), ticarcillin (TIC), ceftriaxone (CRO), tetracycline (TET), and vancomycin (VCA). Notably, CRAB1 had minimum inhibitory concentrations (MICs) of 4 μg/mL for ciprofloxacin (CIP), while CRAB2 and CRAB3 had MICs of 8 μg/mL for this drugs, respectively. These results indicate that these strains exhibit intermediate sensitivity to levofloxacin and ciprofloxacin. Additionally, all strains were highly sensitive to tigecycline (TGC) (MIC <1 μg/mL) (Table 3).

**TABLE 3:** Antibiotic Susceptibility test results (All experiments were conducted with three technical replicates).

Strain ID	CRAB1 MIC (μg/mL)	CRAB2 MIC (μg/mL)	CRAB3 MIC (μg/mL)
Meropenem (MEM)	64	64	64
Imipenem (IPM)	64	64	64
Gentamicin (GEN)	64	64	64
Ticarcillin (TIC)	>64	>64	>64
Ceftriaxone (CRO)	>64	>64	>64
Levofloxacin (LEV)	2	4	4
Ciprofloxacin (CIP)	4	8	8
Tetracycline (TET)	64	64	32
Minocycline (MC)	1	2	1
Tigecycline (TGC)	<1	<1	<1
Colistin (CST)	2	4	4
Vancomycin (VCA)	64	64	64

### The β-lactamase gene carriage status of the two CRAB strains

**FIGURE 3:**
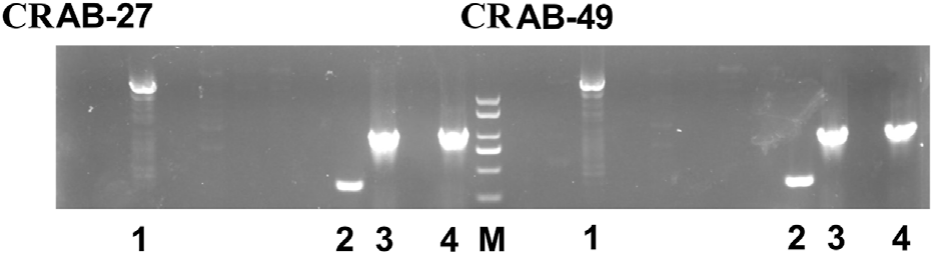
The β-lactamase gene carriage status of CRAB-27 and CRAB-49. (M: Marker; 1: blaVIM; 2: blaOXA-51; 3: blaAmpC; 4: blaOXA-23).

Two CRAB strains (CRAB27 and CRAB49) were collected for analyzing the synergistic effects of carbapenem antibiotics and ciprofloxacin in bactericidal experiments. Prior to the experiments, the β-lactamase gene carriage status of both CRAB strains was analyzed, with the results shown in Figure 3. PCR detection revealed that both CRAB strains carried the blaVIM, blaOXA-51, blaAmpC, and blaOXA-23 genes. The blaVIM gene encodes a Class B metallo-β-lactamase (MBL), while blaOXA-51 and blaOXA-23 encode Class D β-lactamases (OXA enzymes), and blaAmpC encodes a Class C β-lactamase (AmpC enzyme). There were no significant differences in the β-lactamase genotypes between the two CRAB strains, indicating that they likely share similar resistance mechanisms. By confirming the absence of significant genotypic differences between the strains, potential interference from genotypic variations in subsequent resistance mechanism studies can be ruled out. This facilitates a more accurate assessment of the impact of combination therapy on CRAB resistance.

### Checkerboard assay and growth curve

**FIGURE 4:**
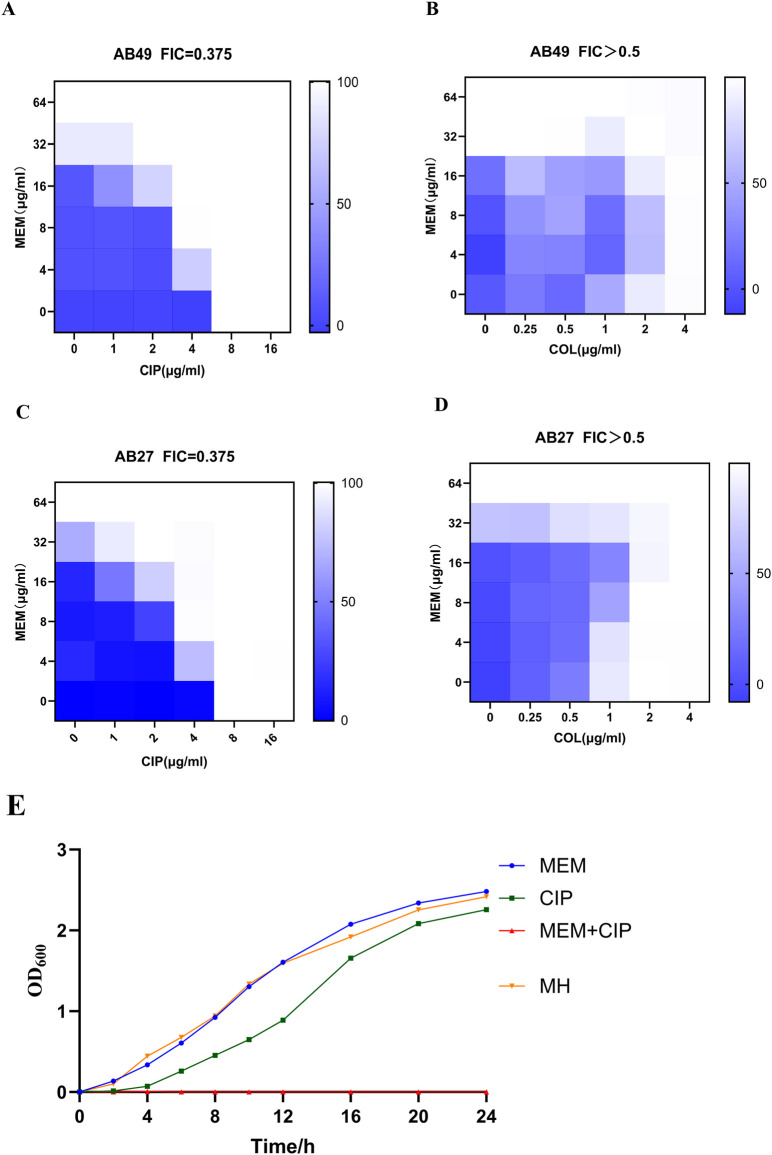
Inhibitory Effects of Different Antibiotics on CRAB. **(A)** Bactericidal effects of MEM with CIP on AB49; **(B)** Bactericidal effects of MEM with COL on AB49; **(C)** Bactericidal effects of MEM with CIP on AB27; **(D)** Bactericidal effects of MEM with COL on AB27; **(E)** Representative growth curves of AB27 (MEM:Meropenem; CIP:Ciprofloxacin; COL:Colistin). Data were presented as mean ± SD from three biological replicates.

The checkerboard assay results indicated that the combination of carbapenem antibiotics and ciprofloxacin exhibited significant synergistic effects against AB27 and AB49. In contrast, the combination of carbapenem antibiotics and colistin showed no interaction against AB27 and AB49. We further used the growth curve of bacteria to describe the bactericidal effects of different antibiotics on CRAB. According to the curve analysis, meropenem alone (blue line) had poor inhibitory effects on the strains, with the OD600 nm value gradually increasing over 24 h, indicating continuous bacterial growth. Ciprofloxacin alone (green line) showed some inhibitory effects compared to meropenem but still failed to completely inhibit bacterial growth. When meropenem was combined with ciprofloxacin (orange line), the OD600 nm value was significantly lower than in other groups within 24 h, indicating a significant bactericidal effect of this combination. In summary, The FICI of MEM combined with CIP is 0.375, indicating that the combination of the two has a synergistic antibacterial effect against CRAB. The FICI of MEM combined with COL is greater than 0.5, indicating that the combination of the two does not have a synergistic effect (Table 4). This demonstrates the potential advantage of the combination therapy of MEM and CIP against CRAB strains (Figure 4).

**TABLE 4:** The FICI values of MEM in combination with two antimicrobial agents (Replications: three times).

Groups	Drug solution	AB27	AB49
Monotherapy MIC	MEM(μg/mL)	64	64
	CIP(μg/mL)	8	8
Combination MIC	MEM(μg/mL)	8	8
	CIP(μg/mL)	2	2
FICI		0.375	0.375

Groups	Drug solution	AB27	AB49
Monotherapy MIC	MEM(μg/mL)	64	64
	COL (μg/mL)	4	4
Combination MIC	MEM(μg/mL)	32	64
	COL (μg/mL)	2	4
FICI		>0.5	>0.5

### The synergy mechanism of meropenem with ciprofloxacin

**FIGURE 5:**
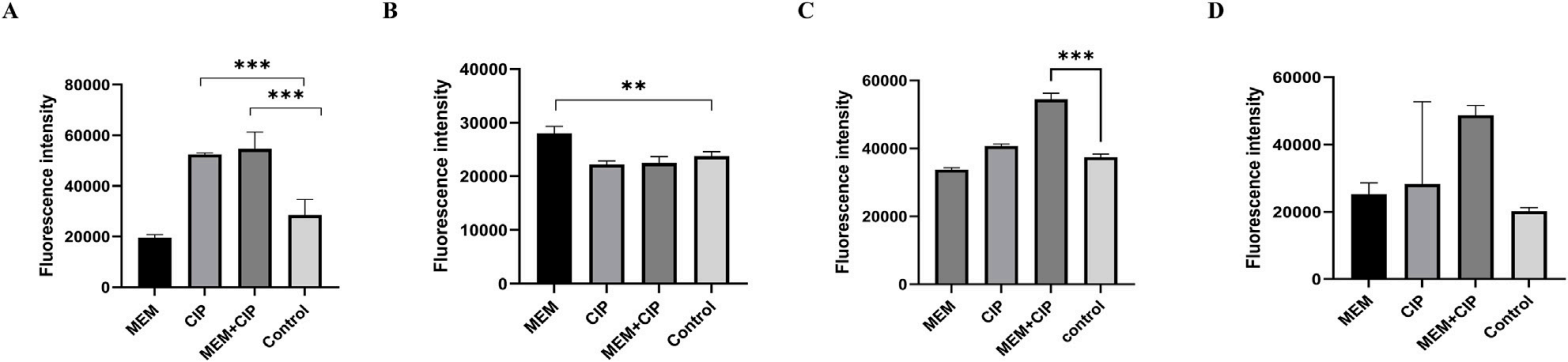
Effects of Different Antibiotics on CRAB. **(A)** Effects of different antibiotic treatments on the outer membrane of CRAB; **(B)** Effects of different antibiotic treatments on the membrane integrity of CRAB; **(C)** Effects of different antibiotic treatments on the membrane potential of CRAB; **(D)** Different antibiotic treatments altered ROS production levels in CRAB (MEM, Meropenem; CIP, Ciprofloxacin). The data are expressed as mean ± SD derived from three biological replicates. The Kruskal–Wallis test was utilized to assess differences in fluorescence alterations among the four groups, followed by the Mann-Whitney U test for pairwise comparisons between groups.

To further elucidate the effects of various antibiotics on CRAB, we examined the impact of different antibiotic treatments on the outer membrane permeability, membrane integrity, membrane potential, and reactive oxygen species (ROS) production of CRAB. The results showed that the combination of meropenem and ciprofloxacin significantly increased the outer membrane permeability of CRAB (*P* < 0.001), with the combined use of meropenem and ciprofloxacin being more effective than either drug alone. Membrane integrity analysis indicated that the meropenem group had significantly higher membrane integrity than other groups (*P* < 0.01). In contrast, the ciprofloxacin alone and combination groups did not show significant differences in membrane integrity but were both lower than the meropenem group. Membrane potential measurements showed that the membrane potential of the meropenem and ciprofloxacin combination group was significantly higher than that of the control group (*P* < 0.05), indicating a significant synergistic effect of this combination in altering membrane potential. In terms of ROS production, both the ciprofloxacin alone and combination groups significantly increased ROS levels, although the combination group showed higher ROS production, the difference was not statistically significant compared to the control group (Figure 5).

### ST typing results of CRAB

In this study, multilocus sequence typing (MLST) was used to analyze the sequence types of carbapenem-resistant *A. baumannii* strains. The results revealed that strains 8, 27, and 49 al l belonged to Sequence Type 208 (ST208). These strains shared identical allele numbers across seven loci: gltA, cnp60, gdhB, gpi, gyrB, rpoD, and recA. This finding suggests that the strains have a common genetic background and likely originated from the same clonal group (Table 5).

**TABLE 5:** ST typing results of CRAB.

Strain	gltA	cnp60	gdhB	gpi	gyrB	rpoD	recA	ST type
8	1	2	3	97	3	3	2	208
27	1	2	3	97	3	3	2	208
49	1	2	3	97	3	3	2	208

### Inhibition of CRAB energy metabolism and efflux activity by meropenem combined with ciprofloxacin

**FIGURE 6:**
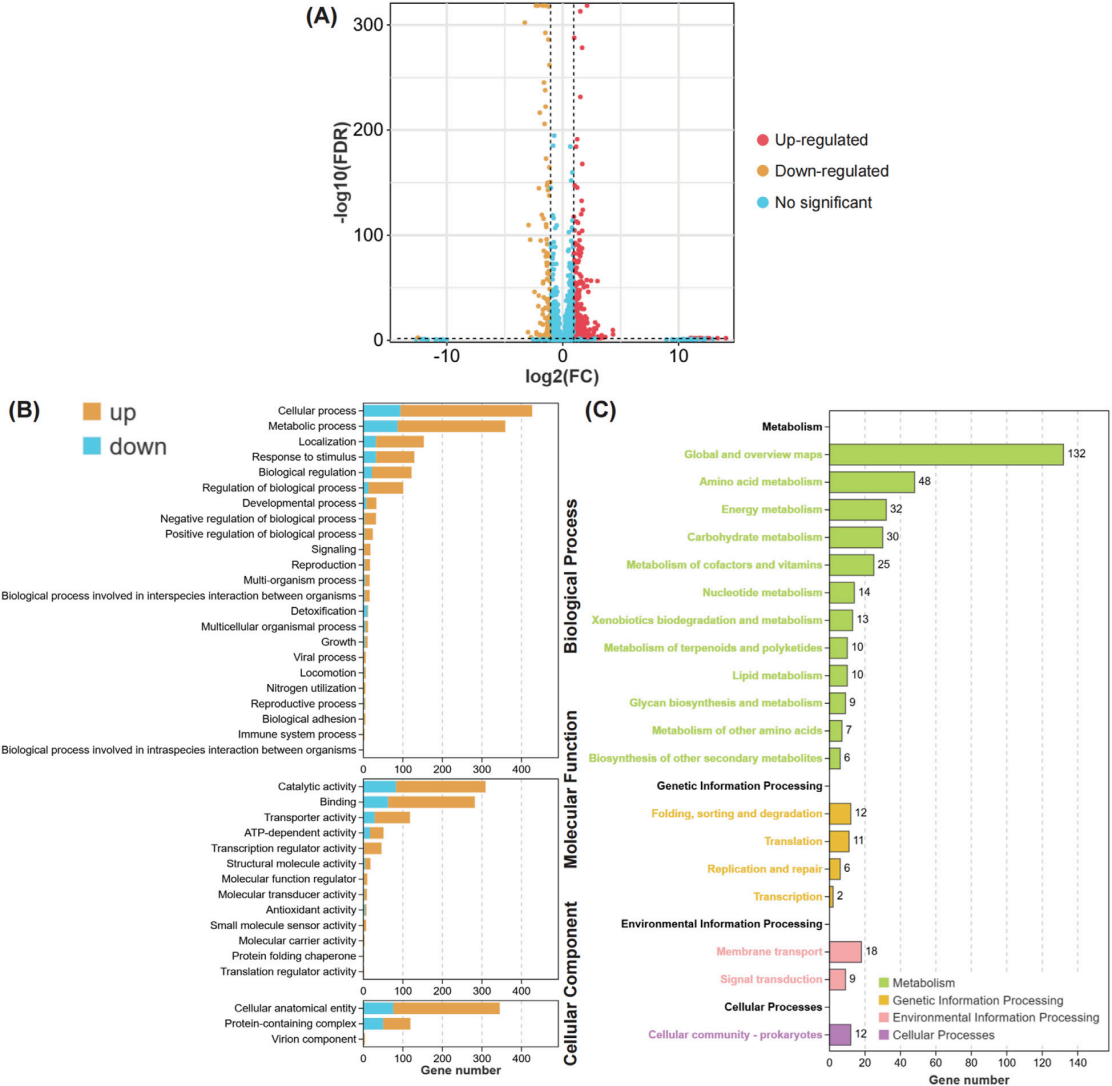
The biological functions of differential expression genes in MIPCEM compared to MH. **(A)** The Volcano plot of differentially expressed genes. The points with different colors represent different alteration trends, Red, Upregulated; Yellow, Downregulated; Blue, No-significant trend. **(B)** The Bar plot of GO enrichment for differentially expressed genes. The figure is divided into three panels based on different types of GO Terms, including biological process, molecular function, and cellular component. The bars in different colors represent the pathway enrichment results for upregulated (Yellow) and downregulated (Blue) genes, respectively. **(C)** The Bar plot of KEGG enrichment for differentially expressed genes. The bar with different colors represent the different types biological pathways, including metabolism (green), genetic information processing (yellow), environmental information processing (fresh pink), and cellular processes (pink).

To investigate the synergistic bactericidal mechanism of ciprofloxacin combined with meropenem against CRAB, the transcriptome of the AB-27 strain was analyzed after ciprofloxacin-meropenem combination treatment. The results showed that, compared to the control group, 480 genes were upregulated and 112 genes were downregulated in CRAB following the combined treatment (Figure 6A). GO enrichment analysis revealed that the differentially expressed genes (DEGs) under meropenem combined with ciprofloxacin treatment (Figure 6B) are primarily associated with the following biological functional categories: Biological Processes: The DEGs are significantly enriched in cellular processes, metabolic processes, localization, and response to stimuli, indicating that the drug treatment has a substantial impact on the basic biological activities of the bacteria. Molecular Functions: The DEGs are predominantly enriched in catalytic activity, binding activity, and transport activity, which are closely related to the bacteria’s metabolic and adaptive responses. Cellular Components: The DEGs are mainly concentrated in cellular components and protein complexes, suggesting that the treatment may induce alterations in the structure and function of the bacterial cell. KEGG pathway analysis further revealed the biological pathways associated with these differentially expressed genes (DEGs) (Figure 6C). The most significantly enriched pathways include: Metabolic Pathways, with the Global and Overview Maps (132 genes) and Amino Acid Metabolism (48 genes) being the most prominently enriched. Other important pathways include Energy Metabolism, Carbohydrate Metabolism, and Cofactor and Vitamin Metabolism, suggesting that the drug treatment may affect bacterial growth and antibiotic resistance by altering metabolic pathways. Genetic Information Processing pathways, such as Folding, Sorting, and Degradation (12 genes) and Transcription (11 genes), indicating that the combined treatment may impact gene expression and protein synthesis in the bacteria. Cellular Processes and Environmental Information Processing pathways were also enriched, highlighting the bacterial adaptive response to environmental changes.

## Discussion

In recent years, *A. baumannii* has become one of the main pathogens of hospital-acquired infections (HAIs). With the widespread use of antibiotics in clinical settings, the resistance rate of *Acinetobacter baumannii* to carbapenem antibiotics has been increasing annually. By 2017, the World Health Organization (WHO) had listed it as a pathogen that poses a significant threat to human health (Boral et al., 2019; Xie et al., 2018). The results of this study indicated that the detection rate of CRAB in our hospital’s ICU, respiratory department, and neurosurgery department is relatively high, at 75.2%, 8.8%, and 8.0%, respectively, which is consistent with previous reports on the distribution of CRAB in clinical departments (Qian et al., 2015). This phenomenon may be related to several factors: first, patients hospitalized in these departments are usually critically ill and have weakened immune systems; second, the routine use of various invasive procedures and long-term broad-spectrum antibiotic treatments. Therefore, medical staff in relevant departments should be vigilant, strengthen environmental hygiene monitoring and disinfection, and strictly follow aseptic procedures to reduce the risk of iatrogenic cross-infection. Additionally, this study shows that CRAB specimens are mainly derived from sputum, which is consistent with previous clinical studies (Lin et al., 2020). This indicates that CRAB primarily causes respiratory infections in our hospital. Therefore, it is particularly important to focus on and strengthen infection prevention and control measures in key departments.

This study shows that the resistance rates of common clinical antibiotics, including β-lactams (excluding fourth-generation cephalosporins), aminoglycosides (excluding tobramycin), and quinolones, all exceed 90%. Additionally, the resistance rate of tetracycline antibiotics is also rising, with the resistance rate of doxycycline exceeding 94%. Kristina et al. (Cerniauskiene et al., 2023) studied the antibiotic resistance of *Acinetobacter baumannii* from 2016 to 2017 and from 2021 to 2022, finding that the proportion of doxycycline-resistant *Acinetobacter baumannii* nearly doubled, which is consistent with the tetracycline resistance rate observed in our study. Furthermore, the resistance rate of sulfonamides is also higher than 50%. Currently, only polymyxin shows 100% intermediate sensitivity to *Acinetobacter baumannii*. However, there have been reports worldwide of the emergence of colistin-resistant *Acinetobacter baumannii*, and its resistance rate is gradually increasing with the widespread use of colistin (CAI et al., 2012). In this study, the resistance rate of *Acinetobacter baumannii* to polymyxin in our hospital is 0%, which differs from the results of Trebosc et al. (2019), possibly due to different antibiotic usage habits in different hospitals. Although *Acinetobacter baumannii* remains sensitive to polymyxin, the emergence of resistance to polymyxin poses a significant challenge to clinical anti-infection treatment. Therefore, developing new treatment strategies remains urgent.

This study found that the proportion of CRAB patients undergoing invasive procedures was significantly higher than that of the AB group. Additionally, CRAB patients had longer hospital stays, ICU stays, mechanical ventilation days, and more types and longer durations of antibiotic use compared to the AB group. CRAB patients also had higher levels of conjugated bilirubin, alanine aminotransferase (ALT), aspartate aminotransferase (AST), and urea, indicating that CRAB patients generally have more severe conditions involving multiple organ dysfunction, leading to a significantly higher incidence of poor prognosis compared to the AB group. Among CRAB patients, those with poor prognosis had higher rates of glucocorticoid use, longer ICU stays, and longer mechanical ventilation days than those with improved conditions. Additionally, patients with poor prognosis had higher white blood cell counts, neutrophil ratios, C-reactive protein (CRP), procalcitonin (PCT), conjugated bilirubin, unconjugated bilirubin, aspartate aminotransferase (AST), urea, creatinine, and creatine kinase isoenzyme (CK-MB) levels than those with improved conditions, which is consistent with previous studies (Borges Duarte and Goncalves Rodrigues, 2022). Therefore, exploring the risk factors and resistance of hospital-acquired infections caused by *Acinetobacter baumannii* and reducing the chances of infection are crucial for early treatment of patients. This study shows that the duration of antibiotic use, application of glucocorticoids, C-reactive protein, and creatine kinase isoenzyme are independent risk factors for poor prognosis in CRAB infection patients. ROC curve analysis shows that C-reactive protein and creatine kinase isoenzyme have certain predictive value for poor prognosis in CRAB patients (AUC = 0.69, AUC = 0.64), which is consistent with previous reports (Zhou et al., 2019). However, the clinical biochemical indicators collected in this study are relatively limited, which is related to the lack of clinical examination data of patients and is one of the limitations of this study. Future studies need to collect a large amount of basic information and clinical biochemical indicators of hospitalized patients infected with *Acinetobacter baumannii* over a long period to further enrich the research results.

*Acinetobacter baumannii* can form complex biofilm structures, allowing it to colonize biological and non-biological surfaces for extended periods. Once a biofilm is formed, the difficulty of treating *Acinetobacter* infections increases significantly, and the risk of transmission between patients also increases, potentially leading to uncontrollable infection outbreaks. Therefore, this study tested the biofilm formation ability of five CRAB strains and selected three strong biofilm-forming CRAB strains for antibiotic susceptibility testing. The results showed that these strains were resistant to all antibiotics except tigecycline, which is consistent with previous reports (Maleki et al., 2022). We performed MLST typing analysis on three CRAB strains, and the results showed that all three strains were ST208. The allele sequences of seven housekeeping genes, gltA, gyrB, gdhB, cpn60, gpi, and rpoD, were identical, while the similarity of the recA gene was 98.92%. This result indicates that ST208 strains are highly conserved, widely distributed, and strongly resistant to carbapenem antibiotics, making them prone to global spread, consistent with the report by Gao et al. (2022) We further conducted antibiotic combination experiments on CRAB strains, and the results showed that the combined use of meropenem and ciprofloxacin could reduce each other’s MIC, indicating a synergistic effect between these two drugs. This result is consistent with the study by Bilal et al. on the combined use of meropenem and ciprofloxacin against *P. aeruginosa* (another Gram-negative *bacillus*) (BILAL et al., 2020). Specifically, the combination of 8 μg/mL meropenem and 4 μg/mL ciprofloxacin had a significant bactericidal effect on carbapenem-resistant *Acinetobacter baumannii*, while the same effect was not achieved when these two drugs were used alone. To our knowledge, this is the first study to report the combined use of meropenem and ciprofloxacin in experiments on carbapenem-resistant *Acinetobacter baumannii* and confirm its significant bactericidal effect.

The resistance of *Acinetobacter baumannii* to carbapenem antibiotics is mainly mediated by the production of β-lactamases, which can catalyze the hydrolysis of β-lactam antibiotics. Based on differences in sequence motifs and hydrolysis mechanisms, β-lactamases can be divided into four classes: A, B, C, and D. Among them, class D β-lactamases, also known as oxacillinases (OXAs) or carbapenem-hydrolyzing class D β-lactamases (CHDLs), can inactivate a broad spectrum of β-lactam antibiotics, especially antibiotics of the OXA-10 family, and have become the main mechanism of carbapenem resistance (Antunes and Fisher, 2014). Bacterial biofilms are the primary means by which *Acinetobacter baumannii* establishes and survives long-term in patients. Additionally, *Acinetobacter baumannii* can persist in hospital environments by forming biofilms. Biofilms create a physical barrier that impedes the penetration of antimicrobial agents; on the other hand, the metabolic activity within biofilms slows down, reducing the bacteria’s response rate to antimicrobial agents, leading to multidrug resistance in *Acinetobacter baumannii* (Qin and Wang, 2019). Efflux pumps are one of the key mechanisms by which bacteria resist the action of antibiotics, actively expelling antibiotics out of bacterial cells to reduce intracellular drug concentrations and prevent them from reaching their intended targets (Ding et al., 2023). The AdeJ gene, belonging to the resistance-nodulation-division (RND) family of efflux pumps, plays a crucial role in this process by expelling multiple antibiotics from bacterial cells, leading to multidrug resistance (MDR) (Zhang et al., 2021). In carbapenem-resistant *Acinetobacter baumannii* (CRAB) strains, the overexpression of AdeJ, along with other efflux pumps such as AdeB and AbeM, is closely associated with high levels of resistance, particularly to carbapenem antibiotics, which are often used as a last resort in clinical treatment (Rafiei et al., 2022). As a significant member of the RND family, AdeJ can expel various antibiotics, including β-lactams, ciprofloxacin, and tetracyclines. Studies have shown that the overexpression of AdeJ in CRAB strains is significantly correlated with their resistance characteristics (Zack et al., 2024). Like most organisms, AB requires iron as an essential element for survival and proliferation during infection (Miethke and Marahiel, 2007). AB secretes siderophores, such as aerobactin, enterobactin, salmochelin, and yersiniabactin, which tightly bind extracellular iron and transport it back into the bacteria (Gonzalez-Ferrer et al., 2021). In this process, ATP-binding cassette (ABC) transporter substrate-binding proteins play a key role by utilizing the energy generated from ATP hydrolysis to transport iron-bound siderophores across the membrane into the cell, a mechanism closely related to AB’s survival in iron-deficient environments (Abdallah et al., 2023). The cysteine ABC transporter substrate-binding protein is part of the ABC transport system, specifically responsible for recognizing and transporting cysteine from the extracellular environment into the cell, which is crucial for bacterial nutrient uptake, metabolic regulation, and adaptation to adverse environmental conditions (Ohtsu et al., 2015). Additionally, it plays an important role in the pathogenicity of pathogens and their ability to colonize the host, making it a potential target for antibacterial drugs (Davies et al., 2021). Our study found that the combined use of ciprofloxacin and meropenem significantly disrupted the integrity of CRAB biofilms, increased membrane permeability, and caused significant membrane depolarization. Ultimately, these changes led to elevated ROS levels within CRAB, triggering oxidative stress responses. Furthermore, our transcriptomic analysis revealed that the combination of ciprofloxacin and meropenem significantly reduced the expression of adeJ and ABC transporter permease in CRAB, effectively inhibiting the function of CRAB efflux pumps and their Fe^2+^ uptake. GO and KEGG enrichment analysis results indicated that the combined treatment of meropenem and ciprofloxacin significantly affected bacterial metabolic pathways, membrane protein synthesis, and stress responses, particularly by regulating metabolism-related genes and pathways, altering the bacteria’s ability to adapt to environmental changes, thereby effectively killing CRAB.

In summary, this study preliminarily explored the bactericidal effect of meropenem combined with ciprofloxacin on CRAB. The results showed that these two drugs have a significant synergistic effect, reducing their respective MICs and enhancing their bactericidal effect. Meropenem is primarily metabolized by the kidneys, so dose adjustments are necessary in patients with renal impairment; ciprofloxacin is metabolized through the blood, kidneys, and bile. However, patients with CRAB infections are often critically ill and frequently suffer from multiple organ failure. In such patients, high-dose medication may further impair organ function. Therefore, a reasonable combination therapy and dose reduction to protect organ function are crucial. The limitation of this study is the small number of strains used, and the specific mechanism of the synergistic bactericidal effect of meropenem and ciprofloxacin was not thoroughly investigated. Future studies will expand the number of strains and delve into the synergistic bactericidal mechanism to provide more reliable theoretical evidence for clinical medication.

## Conclusion

Our findings demonstrate that CRAB patients had more severe clinical outcomes compared to the non-resistant *AB* group, with significantly longer hospital stays, ICU admissions, mechanical ventilation duration, and antibiotic use. Multi-organ dysfunction and higher rates of adverse outcomes were also more prevalent in the CRAB group. Logistic regression identified prolonged antibiotic use, corticosteroid use, elevated C-reactive protein, and creatine kinase isoenzyme levels as independent predictors of poor prognosis. CRAB displayed broad resistance to common antibiotics but remained partially sensitive to tigecycline. The combination of meropenem and ciprofloxacin exhibited synergistic antibacterial effects by lowering the MIC and enhancing bactericidal activity. Certain CRAB strains showed strong biofilm formation, complicating treatment. MLST analysis identified these strains as ST208, suggesting clonal origins. Mechanistically, meropenem and ciprofloxacin disrupted the CRAB outer membrane, increasing permeability, depolarizing membrane potential, and inducing oxidative stress. Transcriptomic analysis revealed inhibition of efflux pump function, reduced iron uptake, and significant disruption of metabolic pathways, membrane protein synthesis, and stress responses, leading to enhanced bacterial clearance. This study underscores the potential of combination therapy in overcoming CRAB resistance and improving treatment outcomes.

## Data Availability

The data obtained have been approved by the Ethics Committee of the Affiliated Hospital of Yangzhou University, and the ethical approval number is 2021-YKL4-28-004. All data are legal and compliant. Due to ethical requirements, the original data cannot be uploaded to public datasets. However, if readers wish to access the data, they may request the information from the corresponding author at 090904@yzu.edu.cn and the raw data supporting the conclusion of this article will be made available by the authors, without undue reservation.
