# Supplementary material for: Synergistic effect and mechanism of meropenem with ciprofloxacin against carbapenem-resistant Acinetobacter baumannii
Source: Front Pharmacol. 2025 May 15;16:1534155. doi: 10.3389/fphar.2025.1534155 (PMC12120557; doi:10.3389/fphar.2025.1534155)
Supplement: Supplementary file 1 [file Table1.docx]

Supplementary Files

**Synergistic Effect and mechanism of meropenem with ciprofloxacin Against Carbapenem-resistant Acinetobacter baumannii**

Ying Feng^a,1^, Xu Chen^a,1^, Yu Sun ^a,1^, Tingting Guo^b^, Feng Wu^a^, Feng Jin^a^, Jun Zhou^a*^

^a^, Department of Respiratory and Critical Care Medicine, Affiliated Hospital of Yangzhou University, Yangzhou University, Yangzhou, China, 225001；

^b^, Department of Microbiology, Medical College, Yangzhou University, Yangzhou， China ,225001）

***** Corresponding Author: Jun Zhou, 090904@yzu.edu.cn

**Suppl. Table S1.** A list of real-time PCR primers design.

**Suppl. Table S2.** A list of distribution of collected CRAB strains used in this study.

**Suppl. Table S3.** A list of clinical antimicrobial susceptibility testing of collected Acinetobacter baumannii.

**Suppl. Table S4.** A list of comparison of clinical characteristics between CRAB and AB. (Non-improvement: Includes patients whose symptoms and clinical indicators did not significantly improve, whose families chose to discontinue treatment due to various subjective factors, and whose symptoms did not improve before discharge, barely maintaining vital signs. Improvement: Patients whose symptoms and signs alleviated after treatment, laboratory test results returned to normal, the condition of the infection site improved, and overall health status enhanced.)

**Suppl. Table S5.** A list of clinical characteristics of poor prognosis in CRAB patients.

**Suppl. Table S1**: A list of real-time PCR primers design.

| Primer Name | Primer Sequence (5’ to 3’) | Product Length (bp) | Annealing Temperature (°C) |
| --- | --- | --- | --- |
| gltA-F | AATTTACAGTGGCACATTAGGTCCC | 722 | 62 |
| gltA-R | GCAGAGATACCAGCAGAGATACACG |  |  |
| gyrB-F | TGAAGGCGGCTTATCTGAGT | 594 | 56 |
| gyrB-R | GCTGGGTCTTTTTCCTGACA |  |  |
| gdhB-F | ACCACATGCTTTGTTATG | 774 | 68 |
| gdhB-R | GTTGGCGTATGTTGTGC |  |  |
| recA-F | CCTGAATCTTCYGGTAAAAC | 425 | 58 |
| recA-R | GTTTCTGGGCTGCCAAACATTAC |  |  |
| cpn60-F | GGTGCTCAACTTGTTCGTGA | 640 | 56 |
| cpn60-R | CACCGAAACCAGGAGCTTTA |  |  |
| gip-F | GAAATTTCCGGAGCTCACAA | 456 | 58 |
| gip-R | TCAGGAGCAATACCCCACTC |  |  |
| rpoD-F | ACCCGTGAAGGTGAAATCAG | 672 | 56 |
| rpoD-R | TTCAGCTGGAGCTTTAGCAAT |  |  |

**Suppl. Table S2**: Distribution of collected CRAB Strains used in this study

| Department | Number of Cases | Proportion |
| --- | --- | --- |
| ICU | 103 | 75.2% |
| Respiratory Medicine | 12 | 8.8% |
| Neurosurgery | 11 | 8.0% |
| General Surgery | 3 | 2.2% |
| Emergency Medicine | 2 | 1.5% |
| Orthopedics | 2 | 1.5% |
| Neurology | 1 | 0.7% |
| Cardiology | 1 | 0.7% |
| Hematology | 1 | 0.7% |
| Oncology | 1 | 0.7% |

**Suppl. Table S3**: Clinical Antimicrobial Susceptibility Testing of collected Acinetobacter baumannii

| Antimicrobial Agents | Drug Name | Resistance Rate(%) | Intermediate Rate（%） | Susceptibility Rate（%） |
| --- | --- | --- | --- | --- |
| β-Lactam Antibiotics | Piperacillin/Tazobactam | 98.54 | 1.46 | 0 |
|  | Cefoperazone/Sulbactam | 95.62 | 2.92 | 1.46 |
|  | Piperacillin | 98.54 | 1.46 | 0 |
|  | Ticarcillin | 98.54 | 1.46 | 0 |
|  | Meropenem | 99.27 | 0 | 0.73 |
|  | Imipenem | 99.27 | 0 | 0.73 |
|  | Ceftazidime (Third Generation) | 96.35 | 1.46 | 2.19 |
|  | Cefuroxime (Third Generation) | 97.81 | 0.73 | 1.46 |
|  | Cefotaxime (Third Generation) | 98.54 | 1.46 | 0 |
|  | Cefepime (Fourth Generation) | 70.80 | 27.74 | 1.46 |
| Aminoglycosides | Tobramycin | 72.99 | 2.19 | 24.82 |
|  | Gentamicin | 97.81 | 2.19 | 0 |
|  | Amikacin | 94.16 | 0.73 | 5.11 |
| Tetracyclines | Tetracycline | 92.70 | 1.46 | 5.84 |
|  | Doxycycline | 94.16 | 0 | 5.84 |
|  | Minocycline | 25.54 | 37.96 | 36.50 |
|  | Tigecycline | 42.33 | 40.88 | 16.79 |
| Quinolones | Nalidixic Acid | 98.54 | 0 | 1.46 |
|  | Norfloxacin | 97.81 | 0 | 2.19 |
|  | Ciprofloxacin | 97.08 | 0.73 | 2.19 |
|  | Levofloxacin | 91.97 | 5.84 | 2.19 |
|  | Moxifloxacin | 96.35 | 2.19 | 1.46 |
| Sulfonamides | Trimethoprim-Sulfamethoxazole | 54.01 | 0 | 45.99 |
| Polypeptides | Polymyxin | 0 | 100 | 0 |

**Suppl. Table S4**: Comparison of Clinical Characteristics Between CRAB and AB

| Clinical Data： | | | | AB  （n=131） | | | | | | CRAB  (n=137） | | | | T/X^2^/Z | | | | | | P Value |
| --- | --- | --- | --- | --- | --- | --- | --- | --- | --- | --- | --- | --- | --- | --- | --- | --- | --- | --- | --- | --- |
| Basic Information： | | | |  | | | |  | | | | | |  | | | |  | | |
|  | Age (years) | | | 70±13 | | | | | 68±15 | | | | | 0.921 | | | | | 0.338 | |
|  | Gender Male | | | 84(64.12%) | | | | | 90(65.69%) | | | | | 0.073 | | | | | 0.788 | |
|  | Female | | | 47(35.88%) | | | | | 47(34.31%) | | | | |  | | | | |  | |
|  | History of Diabetes No | | | 79（60.31%） | | | | | 97（70.80%） | | | | | 3.273 | | | | | 0.070 | |
|  | Yes | | | 52（39.69%） | | | | | 40（29.20%） | | | | |  | | | | |  | |
|  | Invasive Procedures No | | | 20（15.27%） | | | | | 8（5.84%) | | | | | 6.362 | | | | | 0.012 | |
|  | Yes | | | 111（84.73%） | | | | | 129（94.16%） | | | | |  | | | | |  | |
|  | Use of Glucocorticoids No | | | 40（30.53%） | | | | | 29（21.17%) | | | | | 3.073 | | | | | 0.080 | |
|  | Yes | | | 91（69.47%） | | | | | 108（78.83%） | | | | |  | | | | |  | |
| Use of Glucocorticoids No | | 40（30.53%） | | | | 29（21.17%) | | | | | | 3.073 | | | | 0.080 | | | |  |
| Yes | | 91（69.47%） | | | | 108（78.83%） | | | | | |  | | | |  | | | |  |
| Length of Hospital Stay (days) | | 17±9 | | | | 21±11 | | | | | | 8.314 | | | | 0.004 | | | |  |
| Length of ICU Stay (days) | | 5±7 | | | | 13±9 | | | | | | 8.114 | | | | 0.005 | | | |  |
| Duration of Mechanical Ventilation (days) | | 3±6 | | | | 9±8 | | | | | | 15.535 | | | | 0.001 | | | |  |
| Types of Antibiotics Used | | 2±1 | | | | 4±7 | | | | | | 4.362 | | | | 0.038 | | | |  |
| Duration of Antibiotic Use (days) | | 13±7 | | | | 18±9 | | | | | | 7.032 | | | | 0.009 | | | |  |
| Clinical Indicators： | | | | |  | | | | | |  | | | |  | | | | |  |
| White Blood Cell Count（10^9^/L） | | | 10.27±4.41 | | | | 10.73±4.38 | | | | | | 0.304 | | | | 0.592 | | |  |
| Neutrophils（10^9^/L） | | | 8.44±3.71 | | | | 9.34±5.30 | | | | | | 3.450 | | | | 0.062 | | |  |
| C-Reactive Protein（mg/L） | | | 54.42±54.65 | | | | 70.25±62.46 | | | | | | 2.494 | | | | 0.115 | | |  |
| Procalcitonin（ng/L） | | | 1.39±5.01 | | | | 2.16±5.76 | | | | | | 2.414 | | | | 0.121 | | |  |
| Conjugated Bilirubin（umol/L） | | | 3.69±14.68 | | | | 6.32±22.40 | | | | | | 5.444 | | | | 0.020 | | |  |
| Unconjugated Bilirubin（umol/L） | | | 11.56±9.40 | | | | 12.49±9.89 | | | | | | 1.275 | | | | 0.260 | | |  |
| Alanine Aminotransferase（U/L） | | | 23.00（14.00，43.00） | | | | 36.00（26.00，135.00） | | | | | | -3.779 | | | | ＜0.001 | | |  |
| Aspartate Aminotransferase（U/L） | | | 28.00（20.30，45.00） | | | | 41.00（28.00，73.00） | | | | | | -4.364 | | | | ＜0.001 | | |  |
| Glucose（mmol/L） | | | 7.98±3.02 | | | | 8.61±3.30 | | | | | | 0.604 | | | | 0.438 | | |  |
| Urea（mmol/L） | | | 8.41±5.67 | | | | 11.19±7.00 | | | | | | 5.567 | | | | 0.019 | | |  |
| Creatinine（umol/L） | | | 89.93±91.97 | | | | 99.99±85.85 | | | | | | 0.918 | | | | 0.339 | | |  |
| Creatine Kinase（U/L） | | | 67.00（30.00，181.60） | | | | 94.00(30.45，207.50) | | | | | | -0.726 | | | | 0.468 | | |  |
| Creatine Kinase-MB（U/L） | | | 14.93±30.02 | | | | 12.21±17.82 | | | | | | 2.361 | | | | 0.126 | | |  |
| Prognosis Improvement | | | 106（80.92%） | | | | 84（61.31%） | | | | | | 12.471 | | | | ＜0.001 | | |  |
| Non-improvement | | | 25（19.08%） | | | | 53（38.69%） | | | | | |  | | | |  | | |  |

**Suppl. Table S5**: Clinical Characteristics of Poor Prognosis in CRAB Patients

| Clinical Data： | | | Improvement（n=84） | | | | | | Non-improvement(n=53） | | T/X^2^/Z | | | | | | P Value | |
| --- | --- | --- | --- | --- | --- | --- | --- | --- | --- | --- | --- | --- | --- | --- | --- | --- | --- | --- |
| Basic Information： | | |  | | |  | | | | |  | | |  | | | | |
|  | Age (years) | | 66±16 | | | | 70±13 | | | | -1.768 | | | | 0.076 | | | |
|  | Gender Male | | 55(65.48) | | | | 35(66.04) | | | | 0.076 | | | | 0.783 | | | |
|  | Female | | 29(34.52) | | | | 18(33.96) | | | |  | | | |  | | | |
|  | History of Diabetes No | | 63(75.00) | | | | 34(64.15) | | | | 1.850 | | | | 0.174 | | | |
|  | Yes | | 21(25.00) | | | | 19(35.85) | | | |  | | | |  | | | |
|  | Invasive Procedures No | | 6(7.14) | | | | 2(3.77) | | | | 0.671 | | | | 0.413 | | | |
|  | Yes | | 78(92.86) | | | | 51(96.23) | | | |  | | | |  | | | |
|  | Use of Glucocorticoids No | | 9(10.71) | | | | 20(37.74) | | | | 14.219 | | | | ＜0.001 | | | |
|  | Yes | | 75(89.29) | | | | 33(62.26) | | | |  | | | |  | | | |
|  | Length of Hospital Stay (days) | | 22±10 | | | | 19±12 | | | | 1.534 | | | | 0.127 | | | |
|  | Length of ICU Stay (days) | | 11±8 | | | | 15±11 | | | | -2.152 | | | | 0.033 | | | |
|  | Duration of Mechanical Ventilation (days) | | 8±8 | | | | 11±9 | | | | -2.374 | | | | 0.019 | | | |
|  | Types of Antibiotics Used | | 4±9 | | | | 4±2 | | | | 0.198 | | | | 0.843 | | | |
| Duration of Antibiotic Use (days) | | 18±8 | | | 17±10 | | | | | 0.631 | | | 0.529 | | | | |  |
| Clinical Indicators： | | | |  | | | |  | | | |  | | | |  | | |
| White Blood Cell Count（10^9^/L） | | 9.82±3.53 | | | 12.17±5.19 | | | | | -2.895 | | | 0.005 | | | | |  |
| Neutrophils（10^9^/L） | | 8.05±3.20 | | | 11.37±7.00 | | | | | -3.233 | | | ＜0.001 | | | | |  |
| C-Reactive Protein（mg/L） | | 50.09±39.31 | | | 102.19±77.72 | | | | | -4.529 | | | ＜0.001 | | | | |  |
| Procalcitonin（ng/L） | | 0.68±1.64 | | | 4.49±8.57 | | | | | -3.195 | | | ＜0.001 | | | | |  |
| Conjugated Bilirubin（umol/L） | | 1.10±2.67 | | | 14.60±34.45 | | | | | -2.848 | | | 0.006 | | | | |  |
| Unconjugated Bilirubin（umol/L） | | 10.24±6.61 | | | 16.06±12.83 | | | | | -3.056 | | | 0.003 | | | | |  |
| Alanine Aminotransferase（U/L） | | 34.50(20.00,60.75) | | | 36.00(22.00,75.35) | | | | | -0.966 | | | 0.334 | | | | |  |
| Aspartate Aminotransferase（U/L） | | 36.00(26.15,62.23) | | | 53.00(36.00,121.00) | | | | | -3.271 | | | 0.001 | | | | |  |
| Glucose（mmol/L） | | 8.29±2.85 | | | 9.11±3.89 | | | | | -1.423 | | | 0.157 | | | | |  |
| Urea（mmol/L） | | 9.52±5.36 | | | 13.84±8.40 | | | | | -3.347 | | | 0.001 | | | | |  |
| Creatinine（umol/L） | | 83.21±75.63 | | | 126.59±94.72 | | | | | -2.816 | | | 0.006 | | | | |  |
| Creatine Kinase（U/L） | | 92.50(21.50,202.50) | | | 104.00(44.00,238.00) | | | | | -1.133 | | | 0.257 | | | | |  |
| Creatine Kinase-MB（U/L） | | 7.64±6.40 | | | 19.45±26.03 | | | | | -3.241 | | | 0.002 | | | | |  |
|  | Age (years) | | 66±16 | | | | 70±13 | | | | -1.768 | | | | 0.076 | | | |
|  | Gender Male | | 55(65.48) | | | | 35(66.04) | | | | 0.076 | | | | 0.783 | | | |
|  | Female | | 29(34.52) | | | | 18(33.96) | | | |  | | | |  | | | |
|  | History of Diabetes No | | 63(75.00) | | | | 34(64.15) | | | | 1.850 | | | | 0.174 | | | |
|  | Yes | | 21(25.00) | | | | 19(35.85) | | | |  | | | |  | | | |
|  | Invasive Procedures No | | 6(7.14) | | | | 2(3.77) | | | | 0.671 | | | | 0.413 | | | |
|  | Yes | | 78(92.86) | | | | 51(96.23) | | | |  | | | |  | | | |
|  | Use of Glucocorticoids No | | 9(10.71) | | | | 20(37.74) | | | | 14.219 | | | | ＜0.001 | | | |
|  | Yes | | 75(89.29) | | | | 33(62.26) | | | |  | | | |  | | | |
|  | Length of Hospital Stay (days) | | 22±10 | | | | 19±12 | | | | 1.534 | | | | 0.127 | | | |
|  | Length of ICU Stay (days) | | 11±8 | | | | 15±11 | | | | -2.152 | | | | 0.033 | | | |
|  | Duration of Mechanical Ventilation (days) | | 8±8 | | | | 11±9 | | | | -2.374 | | | | 0.019 | | | |
|  | Types of Antibiotics Used | | 4±9 | | | | 4±2 | | | | 0.198 | | | | 0.843 | | | |
